# The C‐terminal segment of collagenase in *Grimontia hollisae* binds collagen to enhance collagenolysis

**DOI:** 10.1002/2211-5463.12510

**Published:** 2018-09-06

**Authors:** Keisuke Tanaka, Naoko Teramura, Osamu Hayashida, Katsumasa Iijima, Teru Okitsu, Shunji Hattori

**Affiliations:** ^1^ Nippi Research Institute of Biomatrix Toride Japan; ^2^ Institute of Industrial Science The University of Tokyo Japan

**Keywords:** bacterial collagenase, *Grimontia hollisae*, metallopeptidase M9 subfamily A, PPC domain, recombinant protein, triple‐helical conformation

## Abstract

The collagenase secreted by *Grimontia hollisae* strain 1706B is a 74 kDa protein that consists of two parts: the catalytic module and a C‐terminal segment that includes the bacterial pre‐peptidase C‐terminal domain. Here, we produced a recombinant C‐terminal segment protein and examined its ability to bind collagen and other characteristics as compared with collagen‐binding domains (CBDs) derived from *Hathewaya histolytica* (*Clostridium histolyticum*) collagenases; these CBDs are the only ones thus far identified in bacterial collagenases. We found that the C‐terminal segment binds to collagen only when the collagen is in its triple‐helical conformation. Moreover, the C‐terminal segment and the CBDs from *H. histolytica* have comparable characteristics, including binding affinity to type I collagen, substrate spectrum, and binding conditions with respect to salt concentration and pH. However, the C‐terminal segment has a completely different primary structure from those of the CBDs from *H. histolytica*. As regards secondary structure, *in silico* prediction indicates that the C‐terminal segment may be homologous to those in CBDs from *H. histolytica*. Furthermore, we performed collagenase assays using fluorescein isothiocyanate‐labeled type I collagen to show that the C‐terminal segment positively contributes to the collagenolytic activity of the 74 kDa collagenase from *G. hollisae*.

**Abbreviations**
CBD: collagen‐binding domain
ColG: *Hathewaya histolytica* type I collagenase
ColH: *Hathewaya histolytica* type II collagenase
FITC: fluorescein isothiocyanate
MMP: matrix metalloproteinase
PPC: bacterial pre‐peptidase C‐terminal domain
QTOF‐MS: quadrupole time‐of‐flight mass spectrometry

*Grimontia hollisae* is a Gram‐negative bacterium that was isolated from clinical samples of patients suffering from acute diarrhea 1; it was once classified in the genus *Vibrio*, but has recently been reclassified in the genus *Grimontia* 2. We have purified the collagenase from *G. hollisae* strain 1706B 3, cloned its gene, and determined its complete nucleotide sequence 4. Subsequently, we deduced its complete amino acid sequence, and found that this collagenase originally possesses a prepro region (aa 1–87), a catalytic module (aa 88–615), and a bacterial pre‐peptidase C‐terminal (PPC) domain (Pfam: PF04151, aa 688–749). Moreover, based on this deduced amino acid sequence, we have classified this collagenase into the metallopeptidase M9 subfamily A (M9A) of the MEROPS database 4. Furthermore, by N‐terminal sequence analysis, we confirmed that this collagenase is secreted as a 74 kDa protein consisting of two parts: the catalytic module and the C‐terminal segment including the PPC domain 4.

*Hathewaya histolytica*, which has been recently reclassified from *Clostridium histolyticum* 5, produces two classes of collagenases that belong to metallopeptidase M9 subfamily B (M9B): *Hathewaya histolytica* type I collagenase (ColG) and *Hathewaya histolytica* type II collagenase (ColH). ColG contains two PPC domains and ColH contains one PPC domain 6. Moreover, the PPC domains from M9B collagenases have been shown to be collagen‐binding domains (CBDs) 7, 8, 9. Even though the collagenases from *G. hollisae* and *H. histolyitica* are classified into different M9 subfamilies, *G. hollisae* collagenase possesses a PPC domain as *H. histolyitica* collagenases do. Both of these facts prompted us to ask whether the PPC domain from *G. hollisae* collagenase also serves as a CBD.

In this study, we hypothesized that the C‐terminal segment including PPC domain from 74 kDa *G. hollisae* collagenase binds to collagen by recognizing its triple‐helical conformation just as the CBDs in *H. histolyitica* collagenases do. To test this hypothesis, we produced a recombinant protein of the C‐terminal segment, and examined its capabilities to bind to native collagen and denatured collagen (gelatin). We also characterized the C‐terminal segment by comparing it with the CBDs from *H. histolyitica* collagenases through binding assays, sequence alignments, secondary structure prediction analysis, and CD analysis. Subsequently, we compared the collagenolytic activities between the *G. hollisae* collagenases with and without the C‐terminal segment to evaluate its contribution to collagenolytic activity of *G. hollisae* collagenase. Furthermore, we compared the amino acid sequence of the C‐terminal segment from *G. hollisae* collagenase with those of the PPCs from other M9A collagenases.

## Materials and methods

### Preparation of recombinant C‐terminal segment derived from *G. hollisae* collagenase

The nucleotide sequence of *G. hollisae* collagenase was deposited in the DDBJ database (AB600550). To express the C‐terminal segment (aa 616–767) including the PPC domain, we followed the manufacturer's instructions, and amplified gene fragments (15.4 kDa, 456 bp) using following primers; forward, 5′‐ GATGACGATGACAAAACCGAGGCGCTGGCGAAG‐3′; reverse, 5′‐CATCCTGTTAAGCTTTTACTGACGACACTGGTT‐3′. The underlined sequences are overlapped with both ends of the insert fragment sequence. All PCRs were conducted using the Expand High Fidelity PCR System (Roche, Mannheim, Germany) to amplify insert fragments. The insert fragments were mixed with the linearized *Brevibacillus* expression vector pBIC2 (Takara Bio, Shiga, Japan), and this mixture was transformed into *Brevibacillus choshinensis* competent cells HPD31‐SP3 (Takara Bio) to construct the expression plasmids by the *Brevibacillus in vivo* cloning method. The *Brevibacillus* transformants were aerobically cultured in TMNm medium (10 g·L^−1^ glucose, 10 g·L^−1^ phytone peptone, 5 g·L^−1^ Ehrlich Bonito extract, 2 g·L^−1^ yeast extract B2, 10 mg·L^−1^ FeSO_4_∙7H_2_O, 10 mg·L^−1^ MnSO_4_∙4H_2_O, 1 mg·L^−1^ ZnSO_4_∙7H_2_O, 50 μg·mL^−1^ neomycin, pH 7.0). After centrifugation, the supernatant was filtered with 0.2 μm membrane filter (Advantec, Tokyo, Japan) and purified with a Ni‐chelating column (cOmplete His‐tag Purification Column, Roche). The column was eluted isocratically with 50 mm Tris/HCl (pH 8.0) containing 500 mm NaCl and 250 mm imidazole. The eluted fraction was concentrated by ultrafiltration with a 5 kDa cutoff (Vivaspin 20, GE Healthcare, Little Chalfont, UK) and dialyzed against 50 mm Tris/HCl buffer (pH 8.0) containing 1 mm CaCl_2_ at 4 °C. Enterokinase (1 U·mL^−1^ reaction volume; Life Technologies, Carlsbad, CA, USA) was used to cleave the His‐tag at 25 °C overnight, and the reaction was stopped by adding proteinase inhibitor, Pefabloc (Roche). This solution was applied onto a Ni‐chelating column to remove the cleaved His‐tag fragment. The recombinant C‐terminal segment passed through the Ni‐chelating column, and the flow‐through fraction was concentrated by ultrafiltration with a 5 kDa cutoff (GE Healthcare). The protein was confirmed by quadrupole time‐of‐flight mass spectrometry (QTOF‐MS) using an maXis II spectrometer (Bruker, Billerica, MA, USA), N‐terminal sequence analysis using a Procise 494 protein sequencer (Applied Biosystems, Foster City, CA, USA), and size exclusion chromatography using ÄKTA system (GE Healthcare) with a Superdex 75 10/300 GL column (GE Healthcare). The protein concentrations were determined by using the UV‐vis method with a NanoDrop 1000 (Thermo Fisher Scientific, Waltham, MA, USA). The purified proteins of the C‐terminal segment were stored at −30 °C for the following experiments.

### SDS/PAGE

SDS/PAGE was carried out on 5% polyacrylamide gel or 4–20% gradient gel (Mini‐PROTEAN TGX Gels, Bio‐Rad, Hercules, CA, USA). After electrophoresis, the gel was stained with 0.25% Coomassie Brilliant Blue R‐250 in 50% methanol and 10% acetic acid, and then destained with 5% methanol and 7.5% acetic acid.

### Binding assay

The collagen‐binding assay was carried out according to the previously reported methods 7. Briefly, binding of the C‐terminal segment to insoluble type I collagen fibers was determined by analyzing the amount of remnant C‐terminal segment after incubation with insoluble type I collagen fibers. First, protein mixture was prepared by putting 0.2 mg·mL^−1^ of the C‐terminal segment and 0.2 mg·mL^−1^ of BSA (Thermo Fisher Scientific) into binding buffer (50 mm Tis/HCl buffer, 0.2 m NaCl, 5 mm CaCl_2_, pH 7.5). Secondly, 5 mg of insoluble type I collagen fibers (Sigma‐Aldrich, St Louis, MO, USA) was incubated in 100 μL of the protein mixture at room temperature for 30 min in a centrifugal filter unit with a pore size of 0.22 μm (Ultrafree‐MC‐GV, Merck Millipore, Burlington, MA, USA). After centrifugation of the filter unit at 10 000 ***g*** for 15 min, the filtrate was analyzed by SDS/PAGE. When necessary, the salt concentration in the binding buffer was modified (0, 0.1, 0.5, or 1.0 m). To alter the pH of the binding buffer, Tis/HCl was replaced with 50 mm MES (pH 6.0), HEPES (pH 7.0) or TAPS (pH 8.0, or 9.0). The effect of calcium ions on the binding of the C‐terminal segment was examined by adding 10 mm EGTA into the binding buffer. On the other hand, binding of the C‐terminal segment to gelatin was determined in the same way as described above for the determination of binding to insoluble type I collagen fibers except by using bovine gelatin‐coupled Sepharose beads (Gelatin‐Sepharose 4B, GE Healthcare) instead of insoluble type I collagen fibers.

### Scatchard analysis

To determine the binding affinity of the C‐terminal segment by Scatchard plot analysis, a collagen‐binding assay was carried out as follows: 2.5 mg of insoluble type I collagen fibers was washed with 400 μL of binding buffer (50 mm Tis/HCl buffer, 0.2 m NaCl, 5 mm CaCl_2_, pH 7.5) and resuspended in 50 μL of the binding buffer containing various concentrations (0.1–2.0 mg·mL^−1^) of the C‐terminal segment with 0.05 mg·mL^−1^ BSA. A calibration curve was constructed for each sample by densitometry using NIH imagej software after SDS/PAGE and was used to quantify their amounts in the filtrates. The results obtained by triplicate assay were analyzed on a Scatchard plot, and the dissociation constant (*K*_d_) and the number of binding sites on insoluble collagen (*B*_max_) were determined by the least‐squares method.

### Preparation of type specific collagen‐coupled beads

Various types of collagen molecules [bovine pepsin‐solubilized type I, II, III, and V collagens (Nippi inc., Tokyo, Japan), human pepsin‐solubilized type IV collagen (Collagen Research Center, Tokyo, Japan)] were coupled to Sepharose beads (NHS‐activated Sepharose^TM^ 4 Fast Flow, GE Healthcare). Briefly, type I, II, III, IV, or V collagen (3 mg·mL^−1^ in 5 mm acetic acid) was mixed with an equal volume of twofold coupling buffer (0.4 m NaHCO_3_, 1 m NaCl, 8% sucrose). Two hundred microliters of CNBr‐activated Sepharose was suspended in 1 mL of 1 mm HCl and incubated for 5 min. The suspension was centrifuged at 500 ***g*** for 1 min to wash the beads, and this washing was repeated three times. Then, the beads were suspended in 666 μL of the collagen solution (final 1.5 mg·mL^−1^ in 0.2 m NaHCO_3_, 0.5 m NaCl, 4% sucrose). The suspension was incubated on a rotary shaker at 4 °C for 2 days. After centrifugation of the suspension, the beads were incubated in 500 μL of 0.1 m Tis/HCl (pH 8.0) for 2 h on a rotary shaker to block all remaining active groups on the beads. The beads were sequentially washed with 500 μL each of 0.1 m Tis/HCl (pH 8.0); 0.1 m sodium acetate, 0.5 m NaCl (pH 4.0), and this washing was repeated three times. Finally, the binding buffer was added to the beads to obtain the 100 μL of final suspension. The suspension was stored at 4 °C for the following experiments.

### Amino acid sequence alignment

Sequence similarity searches between PPC domains within the C‐terminal segment from *G. hollisae* and other bacterial collagenases were performed using the protein blast program of the National Center for Biotechnology Information, and multiple sequence alignment analysis was performed using the clustal omega program 10.

### *In silico* secondary structure prediction

*In silico* secondary structure prediction of the PPC domains from collagenases was performed using the NPS@ structure server 11. For this *in silico* secondary structure prediction, we used the amino acid sequences of the PPC domain (aa 647–767) within the C‐terminal segment from *G. hollisae* collagenase (NCBI accession number: BAK39964), ColG s3a (aa 888–999) and s3b (aa 1007–1118) from *H. histolytica* type I collagenases (NCBI accession number: D87215), and ColH s3 (aa 906–1016) from *H. histolytica* type II collagenases (NCBI accession number: AB014075). To validate the prediction, we used the following five methods jointly: MLRC 12, DSC 13, GOR IV 14, PHD 15 and PREDATOR 16.

### CD analysis

CD spectra were recorded on a J‐805 spectropolarimeter (Jasco, Tokyo, Japan) using a 1‐mm path length quartz cell. Protein concentrations were determined by UV absorption at 280 nm using a NanoDrop 1000 UV‐Vis spectrophotometer. The concentrations of the C‐terminal segment or CBDs from *H. histolytica* were 0.1 mg·mL^−1^. The wavelength scanning experiments were done in 10 mm phosphate, pH 7.5 at 20 °C, and the spectra represent the average of eight scans recorded at a wavelength resolution of 0.1 nm. The CD spectrum data were analyzed by using bestsel software available on the Web (http://bestsel.elte.hu/) to predict the proportion of the contents of secondary structures 17, 18.

### Preparation of recombinant *G. hollisae* collagenases

Recombinant 74 kDa collagenase was produced as previously described, and ~ 60 kDa collagenase was made from 74 kDa collagenase through its autodegradation 4. After separation of the *Brevibacillus* culture medium by a DEAE‐Sepharose column under a gradation of sodium concentrations (0.2–1.1 m NaCl), 74 kDa and ~ 60 kDa collagenases were collected separately, concentrated by ultrafiltration with a 30‐kDa cutoff (Pall, Port Washington, NY, USA) and dialyzed against 50 mm Tis/HCl buffer (pH 7.5) at 4 °C.

### Assay for collagenolytic and gelatinolytic activities

The collagenolytic activity of collagenase was measured using fluorescein isothiocyanate (FITC)‐labeled type I collagen as previously described 4. On the other hand, the gelatinolytic activity of collagenase was measured using heat‐denatured FITC‐collagen (FITC‐gelatin). To measure the gelatinolytic activity, the collagenase solution was mixed with 50 mm Tis/HCl (pH 7.5) containing 0.05% FITC‐gelatin, 0.2 m NaCl and 5 mm CaCl_2_, and incubated at 30 °C for 30 min. After adding EDTA to stop the enzymatic reaction, the degraded FITC‐gelatin fragments were isolated from non‐degraded FITC‐gelatin by adding cold 30% trichloroacetic acid at a final concentration of 15% trichloroacetic acid, and the fluorescence intensity (530 nm *E*_m_/485 nm *E*_x_) of the degraded FITC‐gelatin fragments was measured. One unit of gelatinolytic activity was defined as the amount degrading 1 μg of FITC‐gelatin at 30 °C per min.

### Statistical analysis

All data are presented as mean ± standard deviation. Statistical analyses were performed by Student's *t* test (Microsoft Excel). A value of *P* < 0.05 was considered significant.

## Results

### Recombinant C‐terminal segment from *G. hollisae* collagenase and its conformation‐specific binding to collagen

**Figure 1:**
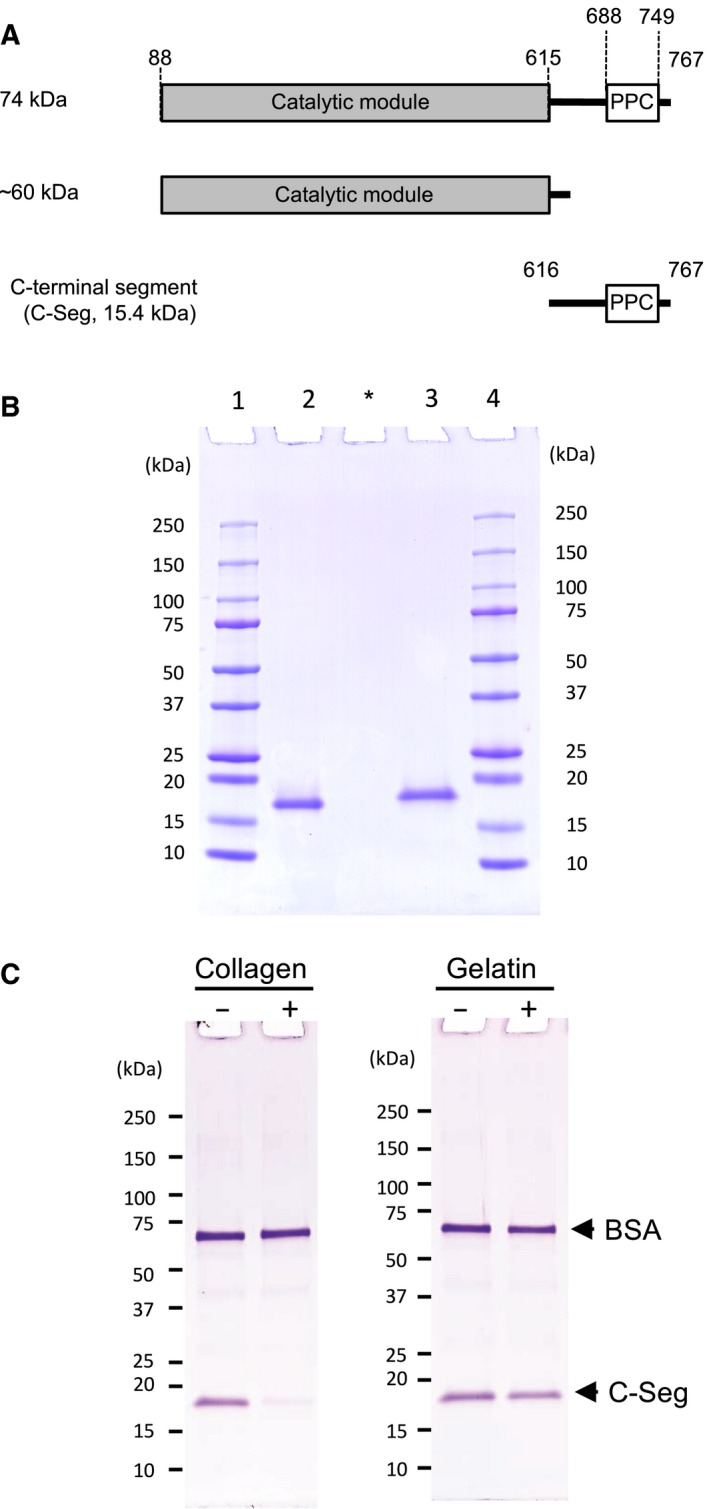
Graphical representation of C‐terminal segment and its conformation‐specific binding to collagen. (A) Recombinant protein was designed as the C‐terminal segment (aa 616–767) including the PPC domain of *G. hollisae* collagenase. (B) The C‐terminal segment was purified from *Brevibacillus* culture medium by affinity chromatography on a Ni column. Two micrograms of purified C‐terminal segment was analyzed by SDS/PAGE using a 4–20% gradient polyacrylamide gel. Lane 1, molecular mass marker; lane 2, C‐terminal segment under non‐reducing conditions; lane 3, C‐terminal segment under reducing conditions; lane 4, molecular mass marker. Asterisk indicates blank lane. (C) Twenty micrograms of C‐terminal segment was incubated in 100 μL of the binding buffer with (+) and without (−) 5 mg of insoluble type I collagen fibers (left panel), or incubated with (+) and without (−) 50 μL of gelatin‐coupled Sepharose beads (right panel). An aliquot (2 μg of protein) of the filtrate was analyzed by SDS/PAGE on a 4–20% gradient gel. Numbers on the left are molecular masses (in kDa) of the markers.

To investigate the ability of the PPC domain from *G. hollisae* collagenase to bind to collagen, we first attempted to recombinantly express the C‐terminal segment including the PPC domain, which consists of residues 616–767 of the full‐length protein (Fig. 1A). Then, we confirmed whether the purified recombinant C‐terminal segment was expressed as we expected. When the C‐terminal segment was analyzed by SDS/PAGE, we found that it was located above the 15 kDa marker as a single band (Fig. 1B). The C‐terminal segment migrates faster under non‐reducing conditions than it does under reducing conditions, indicating that the cysteines in the sequence appear to form intrachain disulfide bonds. QTOF‐MS analysis of the C‐terminal segment showed a molecular mass of 15 445.01 Da (Fig. S1A). On the other hand, the theoretically predicted molecular mass that is deduced from its amino acid sequence is 15 448.72 Da. Considering two disulfide bonds exist in the recombinant protein, the predicted molecular mass in the native state is changed to 15 444.72 Da, which corresponds well to the observed value of 15 445.01 Da. Moreover, we confirmed the first 10 amino acids of the N‐terminal sequence as TEALAKGDSG (Fig. S1B) and found by size exclusion chromatography that the C‐terminal segment exists as a single molecule without aggregation (Fig. S1C). These results clearly show that the C‐terminal segment had been successfully expressed and was ready for the further analysis.

Next, we performed the binding assay of the C‐terminal segment using insoluble type I collagen fibers or gelatin‐coupled Sepharose beads as substrates for the C‐terminal segment. The C‐terminal segment was incubated with and without insoluble type I collagen fibers, and centrifuged. Then, in the SDS/PAGE analysis of the filtrates containing unbound proteins, we found that the amount of the C‐terminal segment apparently decreased when incubated with insoluble type I collagen fibers (Fig. 1C, left panel). In the case of incubating the C‐terminal segment with and without gelatin‐coupled Sepharose beads, the SDS/PAGE analysis of the filtrates showed that the C‐terminal segment remained in the filtrate even after incubation with gelatin‐coupled Sepharose beads (Fig. 1C, right panel). The control protein, BSA, also remained in the filtrates even after incubation with either insoluble type I collagen fibers or gelatin‐coupled Sepharose beads (Fig. 1C). These results clearly show that the C‐terminal segment binds to insoluble type I collagen fibers, but not to gelatin. Moreover, these results indicate that the C‐terminal segment binds to type I collagen by recognizing its triple‐helical conformation.

### Scatchard analysis of collagen binding of C‐terminal segment

**Figure 2:**
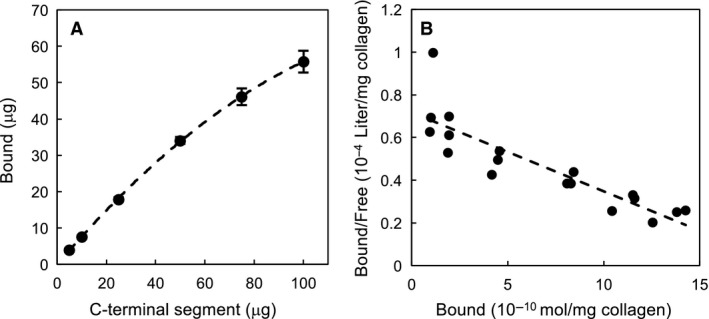
Scatchard analysis of the binding of the C‐terminal segment. Varying concentrations (0.1–2.0 mg·mL^−1^) of the C‐terminal segment were incubated in 50 μL of the binding buffer with a fixed amount (2.5 mg) of insoluble collagen fibers. After incubation, the filtrate containing unbound proteins was subjected to SDS/PAGE, and the amount of the unbound C‐terminal segment was determined by densitometry of the corresponding band. The results obtained by triplicate assay were analyzed on a Scatchard plot. (A) Saturation binding curve for collagen binding of the C‐terminal segment. (B) Scatchard plot for collagen binding of the C‐terminal segment.

To evaluate the affinity of the C‐terminal segment to insoluble type I collagen fibers, we performed collagen‐binding assay by varying concentrations of the C‐terminal segment, and determined the dissociation constant (*K*_d_) as well as the maximum number of binding sites on collagen fibers (*B*_max_). The saturation binding curve demonstrated that the C‐terminal segment binds to collagen fibers in a dose‐dependent manner (Fig. 2A), and a Scatchard plot revealed that the *K*_d_ value is 2.72 ± 0.31 × 10^−5^ m and the *B*_max_ value is 1.94 ± 0.07 nmol·mg^−1^ collagen (Fig. 2B).

### Collagen binding of C‐terminal segment to various types of collagens

**Figure 3:**
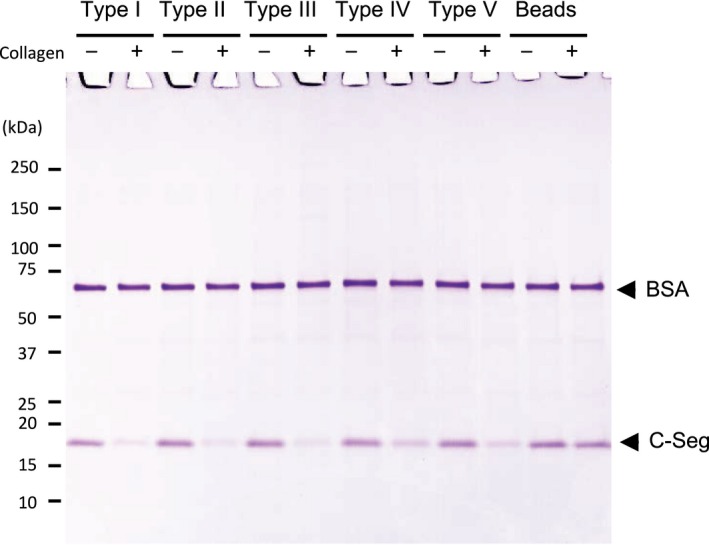
Binding of the C‐terminal segment to various types of collagen. Five micrograms of C‐terminal segment was incubated in 50 μL of the binding buffer with (+) and without (−) various types of collagen coupled‐Sepharose beads (type I, II, III, IV, or V collagen). After incubation, the filtrates were analyzed by SDS/PAGE using a 4–20% gradient polyacrylamide gel. Numbers on the left are molecular masses (in kDa) of the markers.

To investigate whether the C‐terminal segment binds to other types of collagen than type I collagen, a binding assay was performed using type I, II, III, IV or V collagens (Figs 3 and S2). When the C‐terminal segment was incubated with Sepharose beads alone or Sepharose beads coupled with each type of soluble collagen, the C‐terminal segment did not bind to the Sepharose beads alone but bound to all types of collagen‐coupled beads. These results indicate that the C‐terminal segment can bind to not only type I collagen but also other types of collagen such as type II, III, IV, and V collagens.

### Comparative analysis of C‐terminal segment from *G. hollisae* collagenase to CBDs from *H. histolytica* collagenases

The CBDs from *H. histolytica* have been characterized in previous papers 7, 8, 19. To investigate the similarity in primary structure between the PPC domain within the C‐terminal segment from *G. hollisae* and the CBDs from *H. histolytica*, we first performed a multiple sequence alignment using clustal omega 10. We found that the sequence identities between the PPC domain from *G. hollisae* and each of the CBDs from *H. histolytica* (11.24–22.73%) are lower than between the CBDs from *H. histolytica* (30.28–38.53%) (Table 1). These results clearly show that the PPC domain from *G. hollisae* is different in primary structure from the CBDs from *H. histolytica*.

**Table 1:** Percentage identity matrix of the PPC domain from *G. hollisae* collagenase *vs* the CBDs from *H. histolytica* collagenases. The amino acid sequences of PPC domain (aa 647–767) from *G. hollisae* collagenase (NCBI accession number: BAK39964), ColG s3a (aa 888–999) and s3b (aa 1007–1118) from *H. histolytica* type I collagenases (NCBI accession number: D87215), and ColH s3 (aa 906–1016) from *H. histolytica* type II collagenases (NCBI accession number: AB014075) were aligned by clustal omega 10, and the identity matrix was constructed using a multiple protein sequence alignment

	*G. hollisae*	ColG s3b	ColH s3	ColG s3a
*G. hollisae*	100	11.24	21.84	22.73
ColG s3b	11.24	100	30.28	30.63
ColH s3	21.84	30.28	100	38.53
ColG s3a	22.73	30.63	38.53	100

**Figure 4:**
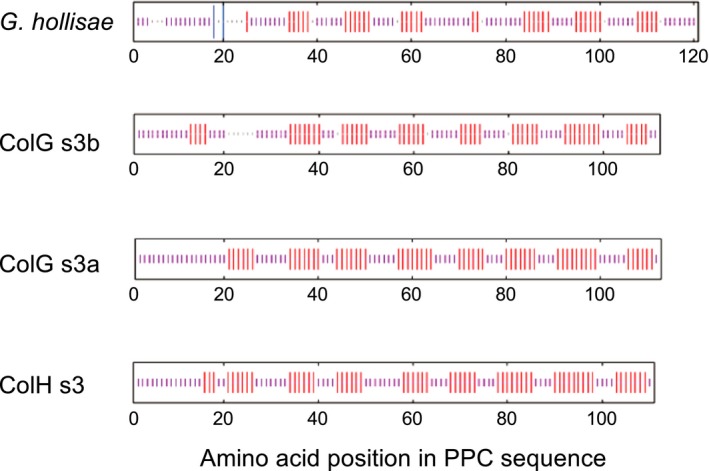
Consensus secondary structures of the PPC domains from *G. hollisae* and *H. histolytica* collagenases. Consensus secondary structures were predicted using a high‐performance method implemented on the NPS@ structure server 11. The predicted structures are represented by colored bars to visualize the schematic architecture: α‐helix, blue; β‐sheet, red; random coil, magenta; unclassified, gray.

To compare the secondary structure of the PPC domain from *G. hollisae* with those of the CBDs from *H. histolytica*, we performed *in silico* secondary structure prediction analysis using the NPS@ structure server 11. This analysis revealed that all of these proteins are mainly composed of β‐sheets and random coils and that they have a similar distribution of β‐sheets (Fig. 4). This result suggests that the C‐terminal segment would be homologous to the CBDs from *H. histolytica* in secondary structure.

**Figure 5:**
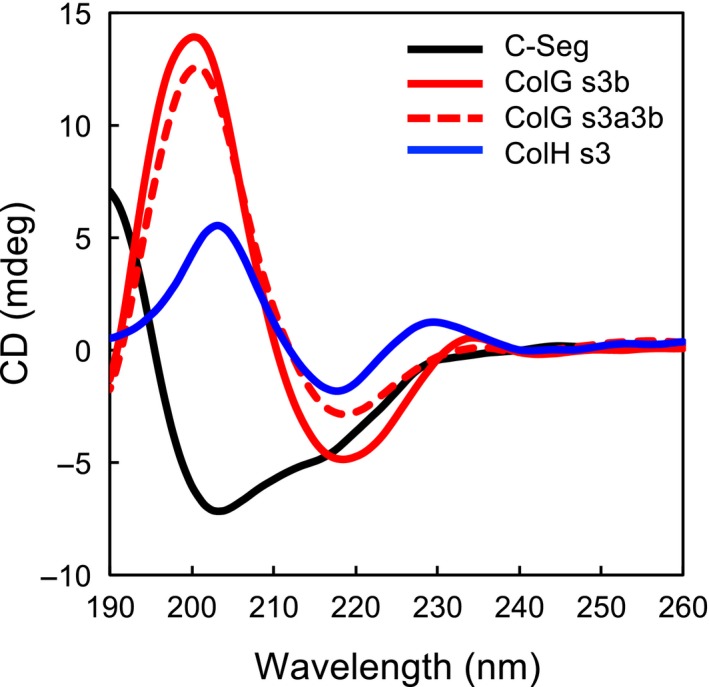
CD spectra of the C‐terminal segment and the CBDs from *H. histolytica*. The CD spectra were measured at 20 °C in 10 mm phosphate, pH 7.5 for the C‐terminal segment (black line), ColG s3b (red solid line), ColG s3a3b (red dashed lines), and ColH s3 (blue line). The concentrations of the C‐terminal segment and the CBDs from *H. histolytica* were 0.1 mg·mL^−1^.

To compare the protein secondary structure content of the C‐terminal segment from *G. hollisae* and those of the CBDs from *H. histolytica*, we measured CD spectra and analyzed those CD spectra data using bestsel software 18. We found that the CD spectrum of the C‐terminal segment and those of the CBDs from *H. histolytica* were completely different (Fig. 5), but they were similar in the percentage of protein secondary structure content (Table 2). We also found that the C‐terminal segment contains more right‐hand twisted antiparallel β‐sheet structure than the three CBDs from *H. histolytica*. We speculate this difference in the proportion of right‐hand twisted antiparallel β‐sheet structure to all β‐sheet structure between the C‐terminal segment and the CBDs from *H. histolytica* causes the inconsistency between the findings in the CD spectra data and the findings in the analysis using bestsel.

**Table 2:** Secondary structure content determined using CD spectra. Single‐spectrum analysis values were calculated using the bestsel method 18

C‐terminal segment	Content (%)				
	α‐Helix	β‐Sheet		Random coil	Turn
		Antiparallel	Parallel		
*G. hollisae*	3.1	42.1 (52.8^a^)	0	42.2	12.6
ColG s3b	2.6	52.4 (26.9^a^)	3.8	34.1	7.1
ColG s3a3b	0	53.2 (28.9^a^)	5.4	33.1	8.3
ColH s3	0	55.6 (36.3^a^)	0	33.0	11.5

a Percentage of right‐hand twisted antiparallel β‐sheet structure of all β‐sheet structure (parallel and antiparallel). Antiparallel β‐sheet is divided into three components: left‐hand twisted, relaxed and right‐hand twisted.

**Figure 6:**
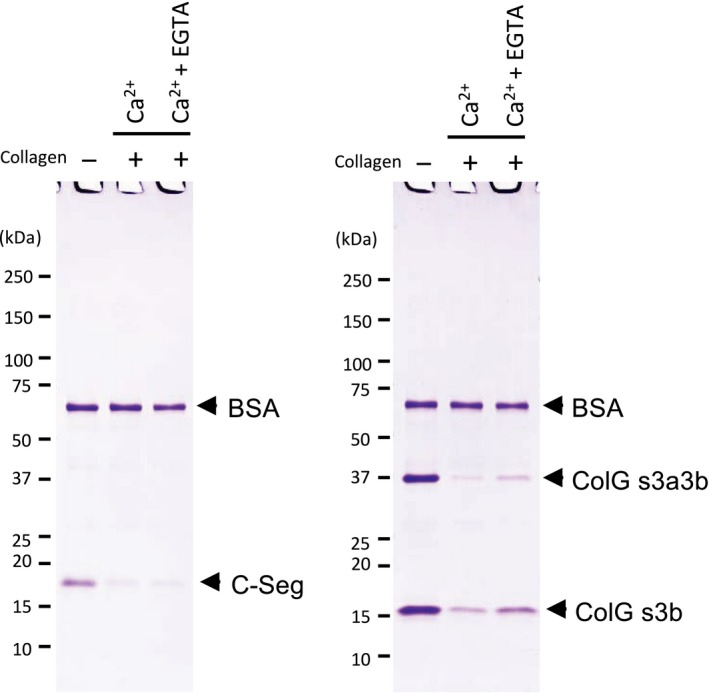
Binding of the C‐terminal segment and ColG CBDs with or without calcium ions. Five micrograms of the C‐terminal segment or 5 μg each of ColG CBDs (s3b and s3a3b) in 50 μL of the binding buffer was incubated with (+) and without (−) 2.5 mg of insoluble type I collagen fibers when varying Ca^2+^ ion concentration (5 mm Ca^2+^ ion or 5 mm Ca^2+^ ion with 10 mm EGTA). After incubation, the filtrates were analyzed by SDS/PAGE using a 4–20% gradient polyacrylamide gel. Numbers on the left are molecular masses (in kDa) of the markers.

Finally, the binding properties of the C‐terminal segment to insoluble type I collagen fibers were analyzed in various buffers. The addition of sodium chloride up to 1 m (Fig. S3A), the alteration of the pH between 6.0 and 9.0 (Fig. S3B), and the addition of 10 mm EGTA (Fig. 6, left panel) did not affect binding of the C‐terminal segment to insoluble type I collagen fibers. However, the addition of 10 mm EGTA inhibited binding of ColG CBDs (s3b and s3a3b) as previously reported (Fig. 6, right panel) 8. These results show that the activation of the C‐terminal segment does not require Ca^2+^, unlike the CBDs from ColG, and suggest that the C‐terminal segment from *G. hollisae* collagenase could bind to collagen with a different collagen‐binding mode compared to the CBDs from *H. histolytica*.

### Effect of C‐terminal segment on collagenolytic and gelatinolytic activities of 74 kDa *G. hollisae* collagenase

**Figure 7:**
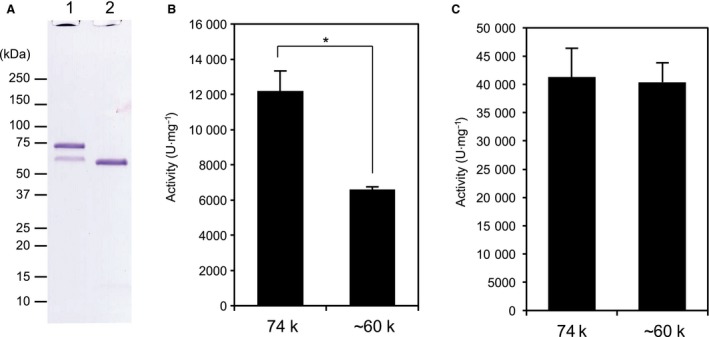
Collagenolytic and gelatinolytic activities of recombinant 74 kDa and ~ 60 kDa *G. hollisae* collagenase. (A) Collagenase of 74 kDa and its truncated form consisting of the catalytic module (~ 60 kDa) were purified from *Brevibacillus* culture medium by DEAE‐Sepharose chromatography. Two micrograms of purified collagenase was analyzed by SDS/PAGE using a 4–20% gradient polyacrylamide gel. Lane 1, 74 kDa collagenase; lane 2, ~ 60 kDa collagenase. Numbers on the left are molecular masses (in kDa) of the markers. (B, C) The collagenolytic and gelatinolytic activities of the recombinant collagenases were determined using FITC‐collagen (B) and FITC‐gelatin (C), respectively. The values represent the average of triplicate trials ± SD. Statistical analyses were performed by Student's *t* test. *P < 0.01.

We previously reported that most of the 74 kDa recombinant collagenase spontaneously becomes truncated to ~ 60 kDa collagenase after it is produced by using the *Brevibacillus* expression system 4. To confirm whether the C‐terminal segment contributes to collagenolytic activity of 74 kDa collagenase, we obtained two types of purified collagenases: 74 and ~ 60 kDa enzymes (Figs 1A and 7A). When the collagenolytic and gelatinolytic activities of both collagenases were examined, we found that the collagenolytic activity of 74 kDa collagenase was almost twice higher than that of ~ 60 kDa collagenase (12 187 ± 1150 *vs* 6599 ± 152 U·mg^−1^, *P *<* *0.01; Fig. 7B). On the other hand, both 74 and ~ 60 kDa collagenases possess comparable degrees of gelatinolytic activity (40 073 ± 3511 *vs* 38 666 ± 3854 U·mg^−1^, *P *=* *0.66; Fig. 7C). These results show that the C‐terminal segment from *G. hollisae* collagenase positively contributes to collagenolytic activity of 74 kDa collagenase.

### Amino acid sequence alignment between the C‐terminal segment from *G. hollisae* collagenase and those from other M9A collagenases

**Figure 8:**
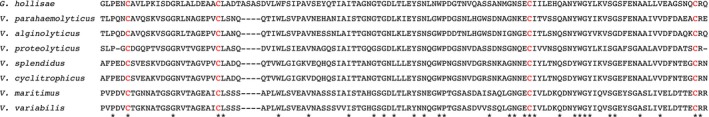
Multiple sequence alignment of the PPC domains from *G. hollisae* collagenase and other M9A collagenases. Multiple sequence alignment of C‐terminal segments from *G. hollisae* collagenase (NCBI accession number: BAK39964, aa 647–767), *Vibrio parahaemolyticus* collagenase (NP_797719, aa 698–814), *V. alginolyticus* collagenase (CAA44501, aa 698–814), *V. proteolyticus* collagenase (WP_021703968, aa 607–721), *V. splendidus* collagenase (WP_102548390, aa 613–729), *V. cyclitrophicus* collagenase (WP_016769033, aa 612–728), *V. maritimus* collagenase (WP_112460283, aa 608–724), and *V. variabilis* collagenase (WP_112477837, aa 608–724) was aided by using the clustal omega program 10. Identical residues among the sequences are indicated by asterisks. Cysteine residues are highlighted in red.

To evaluate similarity between the PPC domain within the C‐terminal segment from *G. hollisae* collagenase and PPC domains from other M9A collagenases, we first searched using the protein blast program for other M9A PPC domains whose primary structures are homologous to the PPC domain within the C‐terminal segment from *G. hollisae*. Next, we performed multiple sequence alignment analysis for the searched M9A PPC domains and the PPC domain from *G. hollisae* by using the clustal omega program 10. We found that seven M9A PPC domains were detected and they possess 41.03–57.26% sequence similarity to the PPC domain from *G. hollisae* (Table S1). These sequence similarities are higher than those between the CBDs from *H. histolytica*; they range from 30.28% to 38.53% (Table 1). Moreover, based on the consideration that disulfide bonds would contribute to the tertiary structure of the PPC domains, we focused on cysteine residues, and found that the seven M9A PPC domains and the PPC domain from *G. hollisae* possess the four cysteine residues that are located at the same positions in the sequences (Fig. 8). These results clearly show that M9A collagenases other than *G. hollisae* collagenase possess PPC domains similar in primary structure to the PPC domain from *G. hollisae* and indicate that they could be homologous to each other to some extent in tertiary structure.

## Discussion

In this study, we have shown that the C‐terminal segment including the PPC domain from 74 kDa *G. hollisae* collagenase is a novel CBD that binds to collagen by recognizing its triple‐helical conformation. Evidence showing that the C‐terminal segment recognizes the triple‐helical conformation of collagen for its binding is that the C‐terminal segment binds to native collagen, but not to denatured collagen (gelatin) (Fig. 1C), since the triple‐helical conformation of collagen exists in the former, but not in the latter. Evidence showing that the C‐terminal segment from *G. hollisae* collagenase is a novel CBD is that the C‐terminal segment differs from any of the three CBDs from *H. histolytica* in primary structure, and the three CBDs from *H. histolytica* are the only ones that have been identified so far in bacterial collagenase. Regarding the primary structure, we found that the sequence identities between the C‐terminal segment and the three CBDs from *H. histolytica* are less than half of the sequence identities between three CBDs from *H. histolytica* (Table 1).

Regarding the higher order structure, we have shown that the PPC domain from *G. hollisae* collagenase and the CBDs from *H. histolytica* could be homologous in secondary structure. This is based on the findings from two methods differing in prediction approach: (a) *in silico* prediction analysis using the NPS@ structure server and (b) CD analysis using bestsel software. Through the former analysis, the PPC domain from *G. hollisae* collagenase and the CBDs from *H. histolytica* was found to be similar in the content as well as the distribution of the secondary structures (Fig. 4). These results for their secondary structure content are supported by the latter analysis (Table 2). Additionally, the CD analysis revealed that the C‐terminal segment possesses a different proportion of antiparallel β‐sheet components compared to the CBDs from *H. histolytica* (Table 2). These different proportions of antiparallel β‐sheet components suggest that the PPC domain from *G. hollisae* collagenase might be different from the CBDs from *H. histolytica* in tertiary structure, although the accurate protein structure will not be clear until it is solved.

Moreover, this study has demonstrated that the C‐terminal segment in 74 kDa *G. hollisae* collagenase facilitates the activity of the catalytic module in degrading collagens specifically when they possess triple‐helical conformation. This is based on the following two findings: (a) ~ 60 kDa *G. hollisae* collagenase consists only of the catalytic module (Fig. 1A), and (b) the collagenolytic activity of 74 kDa collagenase was higher than that of ~ 60 kDa collagenase (Fig. 7B), but the gelatinolytic activity of 74 kDa collagenase was comparable to that of ~ 60 kDa collagenases (Fig. 7C). A characteristic similar to those of the C‐terminal segment has been detected in CBDs from other types of collagenases: a CBD from ColH and a hemopexin domain from matrix metalloproteinase‐1 (MMP‐1) 7, 20. Both *H. histolytica* collagenases and mammalian ones benefit from the presence of evolutionarily unrelated C‐terminal domains to degrade insoluble collagen. The C‐terminal of *H. histolytica* collagenases contains one to two CBDs whereas MMP‐1 consists of a hemopexin domain. The possible role of the C‐terminal segment of *G. hollisae* collagenase in collagenolysis could be to seek a vulnerable region in collagen 21 and to anchor the catalytic module to insoluble collagen 7 or to facilitate active unwinding of collagen as in MMP‐1 20. To clarify this issue, further studies will be needed.

When considering practical applications of the C‐terminal segment from 74 kDa *G. hollisae* collagenase, the segment could be useful as an anchoring molecule in the field of drug delivery. This is because the current study has demonstrated that the C‐terminal segment possesses the binding affinity to various types of collagens under physiological conditions regarding salt concentration and pH (Figs 2, 3 and S3). Since the binding characteristics of the C‐terminal segment are similar to those of the CBDs from *H. histolytica* 7, 8, 19, the C‐terminal segment could be used to deliver drugs and growth factors to blood‐accessible collagen‐rich tissues systemically 22, 23. Also, the C‐terminal segment could be used in conjunction with a collagen‐based scaffold to aid in local delivery of drugs and growth factors 24. In addition, we consider that the applicability of the C‐terminal segment could be expanded to cell culture systems in the field of regenerative medicine. Specifically, any arbitrary functional proteins conjugated to the C‐terminal segment would be immobilized on the collagen‐coated surfaces of Petri dishes. The C‐terminal segment has an advantage in culturing epithelial cells including keratinocytes that need low‐calcium conditions for prevention of their differentiation 25, because it can bind to collagen fibers even under calcium‐free conditions, but the CBDs from *H. histolytica* cannot (Fig. 6).

Furthermore, the identification of a novel CBD from *G. hollisae* collagenase should stimulate basic research into M9A collagenases including elucidation of their collagen recognition and their binding mode toward collagen possessing the triple‐helical conformation. The sequence alignment analysis has detected that some *Vibrio* collagenases are similar not only in primary structure but also in the number and positions of cysteine residues to the PPC domain from *G. hollisae* (Fig. 8). This finding could also encourage both the characterization of the other types of M9A‐PPCs and their structure–function relationship.

In conclusion, we provide evidence that the C‐terminal segment from *G. hollisae* collagenase is a novel CBD and enhances collagenolytic activity of the catalytic module in 74 kDa *G. hollisae* collagenase. The evidence provided in this study will enable the C‐terminal segment to be utilized in applied science and shed light on the basic research into M9A collagenases.

## Author contributions

KT designed the experiments; KT, NT, OH, and KI performed the experiments; KT, NT, OH, KI, TO, and SH analyzed data; KT, TO, and SH wrote the manuscript.

## Conflict of interest

KT, NT, OH, KI, and SH are employees of Nippi, incorporated.

## Supporting information


**Fig. S1.** Characterization of the purified C‐terminal segment. (A) Deconvoluted mass spectrum of C‐terminal segment. The C‐terminal segment desalted with ultrafiltration with a 3 kDa cut‐off (Amicon Ultra) was diluted with 0.1% formic acid/50% acetonitrile and subjected to direct infusion analysis using a QTOF mass spectrometer (maXis II). The obtained mass spectrum was deconvoluted using compass dataanalysis version 4.3 (Bruker Daltonics) with the Maximum Entropy algorithm. (B) Amino acid sequencing chromatogram of C‐terminal segment. The C‐terminal segment was separated by SDS/PAGE and electrophoretically transferred to Immobilon‐P (Merk Millipore). The membrane was stained with Coomassie Brilliant Blue R‐250, and the protein band was excised from the membrane. The N‐terminal sequence was analyzed by using a Procise 491 protein sequencer. The chromatograms of cycle 1–6 are shown. (C) Size exclusion chromatogram of C‐terminal segment. Size exclusion chromatography was performed on an ÄKTA system using Superdex 75 10/300 GL column. The sample was loaded onto a column and eluted isocratically with 20 mm Tis/HCl (pH 7.5) containing 0.2 m NaCl and 1 mm CaCl2 at a flow rate of 0.8 mL·min^−1^. The separated protein fraction was detected at 280 nm. Arrows at the top of the panel indicate void volumes and the apparent molecular mass of standards: BSA, 67 kDa; ovalbumin, 43 kDa; chymotripsinogen A, 25 kDa; and ribonuclease A, 13.7 kDa.
**Fig. S2.** SDS/PAGE analysis of collagens used for preparing collagen‐coupled beads. SDS/PAGE was carried out under reducing condition on 5% polyacrylamide gel for type I, II, III, and V collagen, or 4–20% gradient gel for type IV collagen. After electrophoresis, the gel was visualized by Coomassie Brilliant Blue staining. Numbers on the left are molecular masses (in kDa) of the markers.
**Fig. S3.** Binding of C‐terminal segment in various buffers. Five micrograms each of C‐terminal segment and BSA in 50 μl of reaction buffer was incubated at 25 °C in the absence (−) or presence (+) of 2.5 mg of insoluble type I collagen. After incubation, the filtrates containing unbound proteins were analyzed by SDS/PAGE using a 4–20% gradient polyacrylamide gel. (A) Varying salt concentration. Lanes 1, without NaCl; lanes 2, 0.1 m NaCl; lanes 3, 0.5 m NaCl; lanes 4, 1 m NaCl. (B) Varying pH. Lanes 1, 0.1 m MES (pH 6.0); lanes 2, 0.1 m HEPES (pH 7.0); lanes 3, 0.1 m TAPS (pH 8.0); lanes 4, 0.1 m TAPS (pH 9.0). Numbers on the left are molecular masses (in kDa) of the markers.
**Table S1.** Percentage identity matrix of PPC domains from *G. hollisae* collagenase *vs* other M9A collagenases. The amino acid sequences of PPC domains from *G. hollisae* collagenase (NCBI accession number: BAK39964, aa 647–767), *V. parahaemolyticus* collagenase (NP_797719, aa 698–814), *V. alginolyticus* collagenase (CAA44501, aa 698–814), *V. proteolyticus* collagenase (WP_021703968, aa 607–721), *V. splendidus* collagenase (WP_102548390, aa 613–729), *V. cyclitrophicus* collagenase (WP_016769033, aa 612‐728), *V. maritimus* collagenase (WP_112460283, aa 608‐724) and *V. variabilis* collagenase (WP_112477837, aa 608–724) were aligned by clustal omega program [10], and the identity matrix was constructed using a multiple protein sequence alignment.
