# Supplementary material for: The C‐terminal segment of collagenase in Grimontia hollisae binds collagen to enhance collagenolysis
Source: FEBS Open Bio. 2018 Sep 6;8(10):1691–702. doi: 10.1002/2211-5463.12510 (PMC6168687; doi:10.1002/2211-5463.12510)
Supplement: Supplementary file 1 — Fig. S1. Characterization of the purified C‐terminal segment. (A) Deconvoluted mass spectrum of C‐terminal segment. The C‐terminal segment desalted with ultrafiltration with a 3 kDa cut‐off (Amicon Ultra) was diluted with 0.1% formic acid/50% acetonitrile and subjected to direct infusion analysis using a QTOF mass spectrometer (maXis II). The obtained mass spectrum was deconvoluted using compass dataanalysis version 4.3 (Bruker Daltonics) with the Maximum Entropy algorithm. (B) Amino acid sequencing chromatogram of C‐terminal segment. The C‐terminal segment was separated by SDS/PAGE and electrophoretically transferred to Immobilon‐P (Merk Millipore). The membrane was stained with Coomassie Brilliant Blue R‐250, and the protein band was excised from the membrane. The N‐terminal sequence was analyzed by using a Procise 491 protein sequencer. The chromatograms of cycle 1–6 are shown. (C) Size exclusion chromatogram of C‐terminal segment. Size exclusion chromatography was performed on an ÄKTA system using Superdex 75 10/300 GL column. The sample was loaded onto a column and eluted isocratically with 20 mm Tis/HCl (pH 7.5) containing 0.2 m NaCl and 1 mm CaCl2 at a flow rate of 0.8 mL·min−1. The separated protein fraction was detected at 280 nm. Arrows at the top of the panel indicate void volumes and the apparent molecular mass of standards: BSA, 67 kDa; ovalbumin, 43 kDa; chymotripsinogen A, 25 kDa; and ribonuclease A, 13.7 kDa. Fig. S2. SDS/PAGE analysis of collagens used for preparing collagen‐coupled beads. SDS/PAGE was carried out under reducing condition on 5% polyacrylamide gel for type I, II, III, and V collagen, or 4–20% gradient gel for type IV collagen. After electrophoresis, the gel was visualized by Coomassie Brilliant Blue staining. Numbers on the left are molecular masses (in kDa) of the markers. Fig. S3. Binding of C‐terminal segment in various buffers. Five micrograms each of C‐terminal segment and BSA in 50 μl of reaction buffer was incubated at 25 °C i [file FEB4-8-1691-s001.pdf]

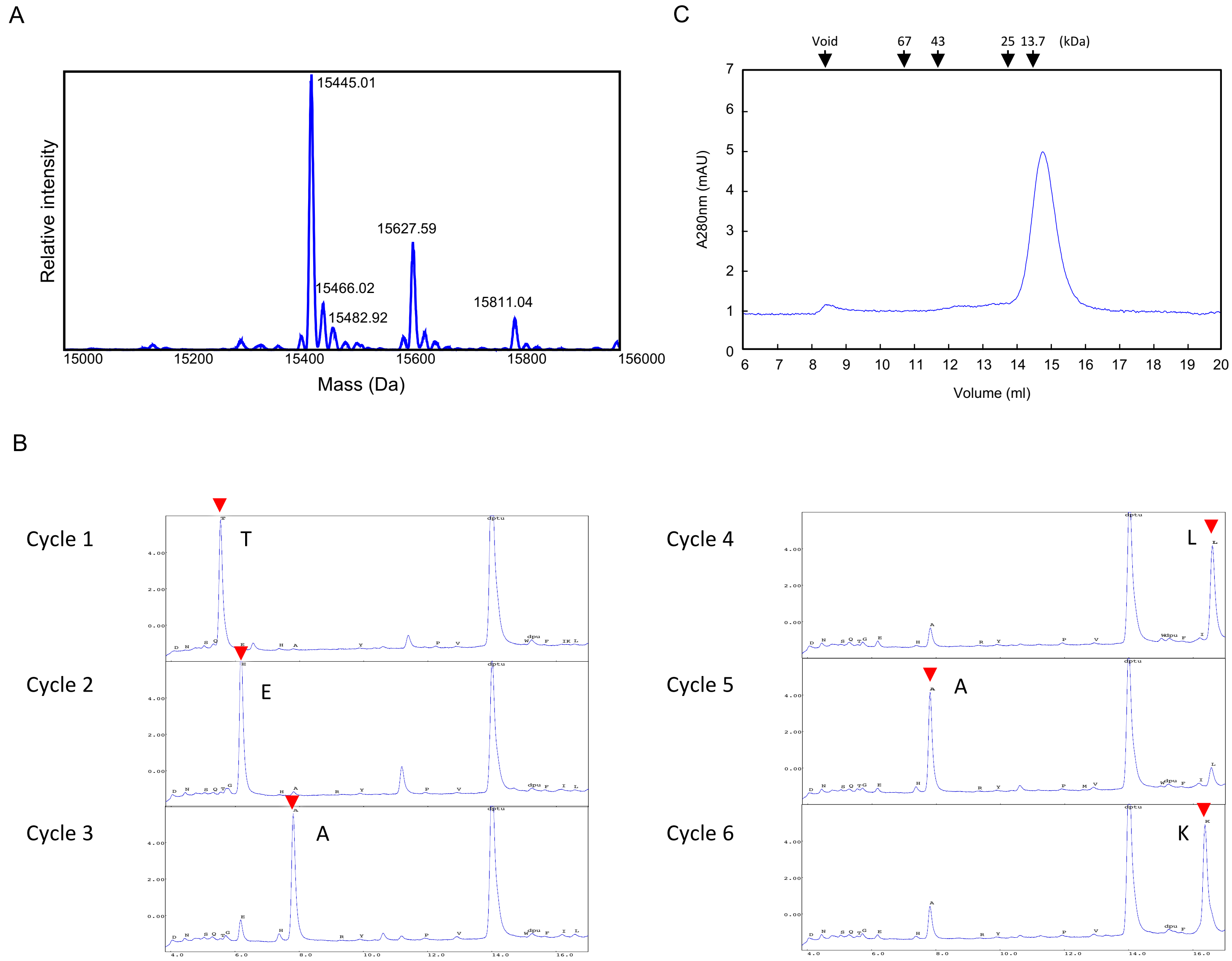

Figure S1 Characterization of the purified C-terminal segment.

(A) Deconvoluted mass spectrum of C-terminal segment. The C-terminal segment desalted with ultrafiltration with a 3 kDa cutoff (Amicon Ultra) was diluted with 0.1% formic acid/50% acetonitrile and subjected to direct infusion analysis using a QTOF mass spectrometer (maXis II). The obtained mass spectrum was deconvoluted using COMPASS DATAANALYSIS version 4.3 (Bruker Daltonics) with the Maximum Entropy algorithm.

(B) Amino acid sequencing chromatogram of C-terminal segment. The C-terminal segment was separated by SDS/PAGE and electrophoretically transferred to Immobilon-P (Merk Millipore). The membrane was stained with Coomassie Brilliant Blue R-250, and the protein band was excised from the membrane. The N-terminal sequence was analyzed by using a Procise 491 protein sequencer. The chromatograms of cycle 1–6 are shown.

(C) Size exclusion chromatogram of C-terminal segment. Size exclusion chromatography was performed on an ÄKTA system using Superdex 75 10/300 GL column. The sample was loaded onto a column and eluted isocratically with 20 mM Tris/HCl (pH 7.5) containing 0.2 M NaCl and 1mM $CaCl_2$ at a flow rate of 0.8 mL·min^{-1}. The separated protein fraction was detected at 280 nm. Arrows at the top of the panel indicate void volumes and the apparent molecular mass of standards: BSA, 67 kDa; ovalbumin, 43 kDa; chymotripsinogen A, 25 kDa; and ribonuclease A, 13.7 kDa.

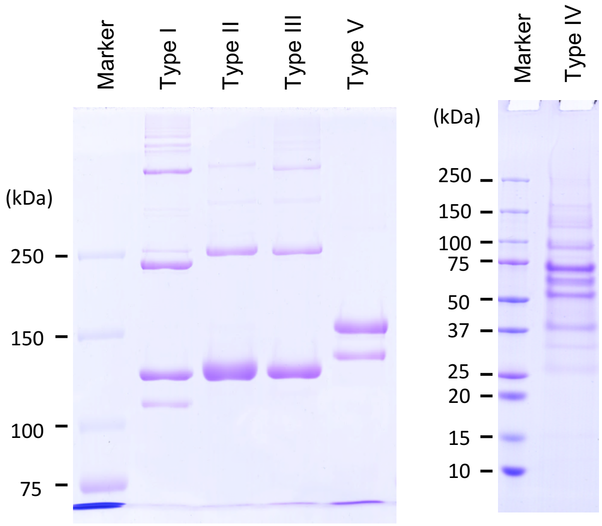

Figure S2 SDS/PAGE analysis of collagens used for preparing collagen-coupled beads.

SDS/PAGE was carried out under reducing condition on 5% polyacrylamide gel for type I, II, III and V collagen, or 4–20% gradient gel for type IV collagen. After electrophoresis, the gel was visualized by Coomassie Brilliant Blue staining. Numbers on the left are molecular masses (in kDa) of the markers.

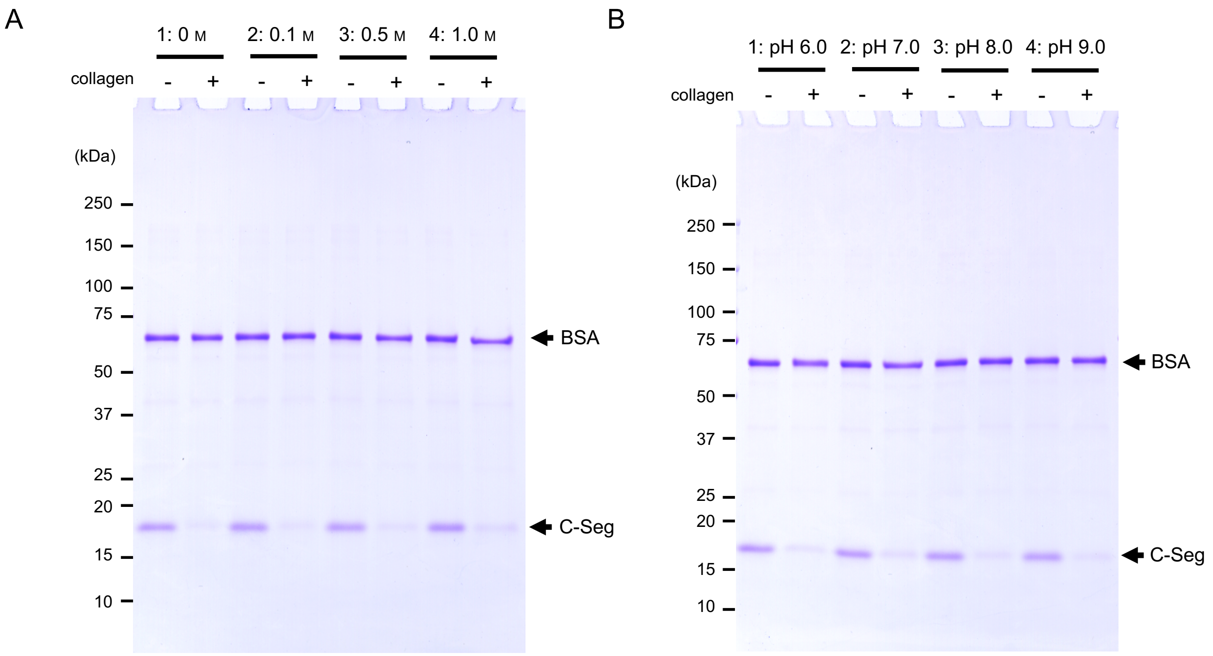

Figure S3 Binding of C-terminal segment in various buffers.

Five micrograms each of C-terminal segment and BSA in 50 μl of reaction buffer was incubated at 25 °C in the absence (-) or presence (+) of 2.5 mg of insoluble type I collagen. After incubation, the filtrates containing unbound proteins were analyzed by SDS/PAGE using a 4–20% gradient polyacrylamide gel. (A) Varying salt concentration. Lanes 1, without NaCl; lanes 2, 0.1 M NaCl; lanes 3, 0.5 M NaCl; lanes 4, 1 M NaCl. (B) Varying pH. Lanes 1, 50 mM MES (pH 6.0); lanes 2, 50 mM HEPES (pH 7.0); lanes 3, 50 mM TAPS (pH 8.0); lanes 4, 50 mM TAPS (pH 9.0). Numbers on the left are molecular masses (in kDa) of the markers.

Table S1. Percentage identity matrix of PPC domains from *G. hollisae* collagenase vs other M9A collagenases.

The amino acid sequences of PPC domains from *G. hollisae* collagenase (NCBI accession number: BAK39964, aa 647–767), *V. parahaemolyticus* collagenase (NP_797719, aa 698–814), *V. alginolyticus* collagenase (CAA44501, aa 698–814), *V. proteolyticus* collagenase (WP_021703968, aa 607–721), *V. splendidus* collagenase (WP_102548390, aa 613–729), *V. cyclitrophicus* collagenase (WP_016769033, aa 612–728), *V. maritimus* collagenase (WP_112460283, aa 608–724) and *V. variabilis* collagenase (WP_112477837, aa 608–724) were aligned by CLUSTAL OMEGA program, and the identity matrix was constructed using a multiple protein sequence alignment.

	V. maritimus	*V. variabilis*	*G. hollisae*	*V. splendidus*	*V. cyclitrophicus*	*V. proteolyticus*	*V. parahaemoluticus*	*V. alginolyticus*
V. maritimus	100	92.31	44.44	46.15	46.15	55.65	47.01	46.15
V. variabilis	92.31	100	41.30	43.59	43.59	53.91	45.30	45.30
G. hollisae	44.44	41.03	100	49.57	49.57	54.78	57.26	56.41
V. splendidus	46.15	43.59	49.57	100	98.29	56.52	59.83	61.54
V. cyclitrophicus	46.15	43.59	49.57	98.29	100	56.52	58.97	60.68
V. proteolyticus	55.65	53.91	54.78	56.52	56.52	100	61.74	60.87
V. parahaemoluticus	47.01	45.30	57.26	59.83	58.97	61.74	100	81.20
V. alginolyticus	46.15	45.30	56.41	61.54	60.68	60.87	81.20	100
